# Alteration of Sulfur-Bearing Silicate–Phosphate (Agri)Glasses in Soil Environment: Chemical Interactions and Biological Response

**DOI:** 10.3390/molecules30081790

**Published:** 2025-04-16

**Authors:** Anna Berezicka, Agnieszka Wojteczko, Justyna Sułowska, Magdalena Szumera

**Affiliations:** Faculty of Materials Science and Ceramics, AGH University of Krakow, al. A. Mickiewicza 30, 30-059 Krakow, Poland; agdudek@agh.edu.pl (A.W.); sulowska@agh.edu.pl (J.S.)

**Keywords:** glass–soil interactions, nutrient-bearing glasses, ion exchange, sulfate incorporation, fertilizer, soil incubation experiment

## Abstract

Glasses exposed to soil environments are of interest across various scientific fields, from nuclear waste containment to archaeological preservation and nutrient-delivery systems for plants. While immersion experiments provide valuable insights into the ion release kinetics in root- and microbe-exuded solutions, they fail to replicate the complexities of nutrient leaching in real soil conditions. To address this, the degradation behavior of nutrient-bearing glasses (41SiO_2_·6(10)P_2_O_5_·20K_2_O·33(29)MgO/CaO/MgO + CaO) with increasing sulfate contents was investigated through a soil incubation experiment simulating Central European weather variability. A comprehensive approach, combining SEM observations and EDS semi-quantitative analysis, revealed that acidic peat strongly promoted ion exchange, where protons from the medium replaced network cations. The glass composition played a crucial role in the fracture behavior: sulfate incorporation increased the network rigidity, making the glasses more prone to mechanical degradation and accelerating the reaction front advancement. The P_2_O_5_ content was also a key factor in modulating the reactivity, with higher concentrations intensifying interactions with the soil medium. Limited water availability accelerated the solution saturation, leading to secondary phase precipitation and temporary nutrient immobilization. These findings demonstrate that glass reactivity can be fine-tuned through composition adjustments and highlight the dynamic nature of glass–soil interactions, including seasonal variations in nutrient release under acidic conditions.

## 1. Introduction

Despite the unprecedented level of understanding of the amorphous state, glass still poses a challenge to scientists at both the microscopic and thermodynamic levels. Microscopically, its random network lacks the long-range order characteristic of crystalline solids, while thermodynamically, it is a nonequilibrium material that undergoes slow structural relaxation, progressively reaching lower free-energy minima [1]. This intrinsic tendency to minimize free energy makes glass susceptible to irreversible transformation upon contact with liquid media, potentially leading to degradation over time [2,3]. Such alterations may be imperceptibly slow [4], but according to Grambow’s hypothesis [5], they never cease due to the metastable nature of the material. The resistance of glass to this irreversible damage and the mechanisms governing its alteration are critical for numerous fields, starting from applications requiring superior chemical durability, such as nuclear waste storage, cultural heritage preservation, and optical fibers, where moisture-induced degradation can severely impact the performance, to applications where controlled dissolution is desirable, like in the case of biomedical glasses for bone regeneration [6,7,8,9,10], or, significantly less studied, vitreous fertilizers—materials designed to supply plants with a variety of macro- and microelements essential for optimal growth and development [11,12,13,14,15,16].

Interestingly, despite extensive research on glass corrosion, efforts to develop a universal model describing the interactions and reactions between aqueous solutions and vitreous materials have failed, incapable of capturing the vast variability in glass compositions, structures, bonding characteristics, and thermal histories, as well as the wide range of environmental factors influencing the alteration process [3,17,18,19]. Indeed, the macroscopic response of a glass in contact with a corrosive medium arises from the aggregation of numerous atomistic-level mechanisms, encompassing species originating from both the solution and the glass itself. Nevertheless, the commonly proposed mechanisms of glass alteration share fundamental steps that, at the molecular level, proceed in a similar manner for all silicate-based glasses. These sequential stages include (1) the adsorption of water onto the glass surface, (2) the simultaneous occurrence of three competing reactions: hydration, hydrolysis, and ion exchange, and (3), in some cases, the precipitation of secondary phases on the altered glass surface [18,20,21,22,23].

Expanding on this simplified mechanism of glass interaction with the contacting solution, shortly after the initial water molecules adhere to the glass’s outermost regions, they interact with adsorption sites, leading to the breakage of surface chemical bonds and the formation of a hydroxylated surface. This process is followed by the penetration of water into the glassy matrix—a step that marks the onset of irreversible material degradation. In glasses with loosely structured vitreous networks, characterized by the presence of sufficiently large openings that facilitate molecular water infiltration, hydration occurs, allowing H_2_O to enter the glass as an intact solvent. Conversely, in glasses with highly interconnected networks, where water penetration is hindered, the glass–solvent interaction is predominantly limited to hydrolysis, involving the cleavage of Si-O-Si backbone-forming linkages in favor of Si-OH bonds, and gradually leading to network depolymerization. The third mechanism, ion exchange (or interdiffusion), entails the replacement of network modifiers by H^+^/H_3_O^+^ ions from the surrounding solution and results in the depletion of these elements in the affected area. As a consequence of these processes, a silica-rich alteration layer forms at the interface between the glass and the corrosive medium, with its morphology and composition strongly influenced by both the intrinsic properties of the glass and the environmental conditions. Under specific circumstances that promote the accumulation of a thin interfacial film of solution supersaturated with respect to leached mobile glass components, the precipitation of secondary phases may occur at the outermost region of the altered glass—a phenomenon known to affect the further evolution of interactions at the glass–solution interface [7,8,10,23,24,25].

The aforementioned processes are highly nuanced, with their progression best elucidated in the context of glasses undergoing corrosion immersed in aqueous environments. In contrast, significantly less research has been devoted to amorphous materials exposed to soil media. This disparity is unsurprising, given the challenges of analyzing glass degradation in this biotic system, where multiple variables—such as the soil composition, moisture fluctuations, temperature changes, pH variations, and chemical diversity—interact in intricate ways [26,27,28,29,30].

The complexity and interconnected nature of soil corrosion-affecting parameters make the process of glass alteration difficult to fully comprehend. In acidic conditions, degradation primarily occurs through ion exchange, leading to an alkali-depleted surface and, in some cases, the formation of a hydrated gel that temporarily slows further alteration [3,4]. According to Palomar [31], such glasses often exhibit networks of isolated or interconnected fissures on their surfaces. Conversely, alkaline soils induce a more aggressive attack by the corrosive medium, manifesting itself through the breakdown of the Si-O-Si-based vitreous network and the removal of silica into the adjacent solution. In this case, the outermost glass layer may assume the form of either a very thin alteration crust [31] or finely laminated layers [3,4,32,33,34]. Numerous studies indicate that the prevalent phenomenon of the degradation of vitreous samples subjected to moist soil is the development of hydrated dealkalinization layers enriched with silica [3,4]. In contrast, when water availability is low, the removal of modifier cations from the glass matrix causes a rapid increase in the pH of the contact solution, subsequently accelerating the deterioration of the silicon–oxygen backbone [35]. Under frequent rainfall conditions, these processes occur differently: the leached ions are washed away from the subsurface area, maintaining a near-neutral pH and preventing the onset of alkaline-driven corrosion [35]. Additionally, alternating rain and drought cycles, along with seasonal and daily temperature fluctuations, significantly influence glass weathering, since not only do they affect ion exchange, but they also promote the formation of secondary corrosion products and contribute to shrinkage-induced cracking in the alteration zone [3,35,36,37,38].

Beyond soil environment-related factors influencing glass deterioration, another crucial aspect is its composition and, consequently, its structure. Indeed, it is well established that a low-silica, alkali-rich composition results in an open network—a feature of utmost importance for enhanced water penetration. And while the durability of silicate-based glasses inversely correlates with the quantity of alkali network modifiers, the same is not necessarily true for alkaline-earth oxide cations. In fact, glass resistance to corrosive media improves with their addition, provided it does not exceed approximately 15 mol.% [4]. Interestingly, the incorporation of a second network former, such as P_2_O_5_, does not always enhance the durability of silicate–phosphate glasses. Conversely, it has been found that unless the P_2_O_5_ content is sufficient to facilitate the formation of a highly connected phosphorus–oxygen subnetwork, an increase in the [PO_4_^3−^] species concentration—when present as isolated or corner-connected tetrahedra—not only fails to reinforce the glass structure but also increases the glass reactivity [4,6,39].

Also of great significance in glass–soil interactions is the role of additives and trace elements, as even minor compositional variations can lead to substantial differences in durability. Among these, sulfur is particularly noteworthy—a component rarely incorporated into the vitreous framework but increasingly relevant not only in modern glass technology but also in plant nutrition, as, due to the significant reduction in SO_2_ emissions into the atmosphere, the primary sulfur source in the rhizosphere has declined [40,41,42,43,44,45,46]. Given that, to the best of the authors’ knowledge, no studies have examined the chemical behavior of sulfate-bearing glasses in soil environments, the present study aims to address this knowledge gap by providing integrated insights into the alteration of sulfur-bearing multicomponent silicate–phosphate glasses.

## 2. Results and Discussion

### 2.1. Surface Alteration Trends Across the Studied Glass Compositions

This section aims to provide scientific insight into the weathering mechanisms of glass fertilizers under environmental conditions relevant to their intended application. Understanding these processes is essential for evaluating how glass–soil interactions influence the release of macro- and microelements. Given its ability to capture both chemical and physical transformations at the material’s surface, SEM-EDS was selected as the most effective technique for monitoring such interactions throughout the incubation process.

**Figure 1:**
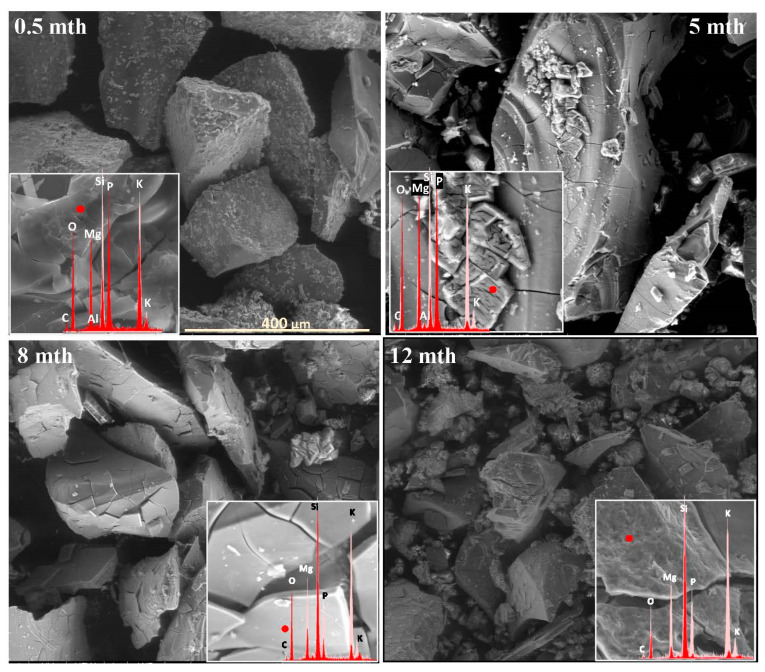
Micrographs illustrating surface changes in the 0S_6PM glass after 0.5, 5, 8, and 12 months of incubation, accompanied by EDS spectra from selected surface points (red spectra) compared with the spectrum of the unaltered glass (light-pink spectrum). The main microphotographs were taken at 350× magnification, while the inset images were captured at 5000×. The scale bar shown in the first image (400 μm) applies to all other images in the figure.

**Figure 2:**
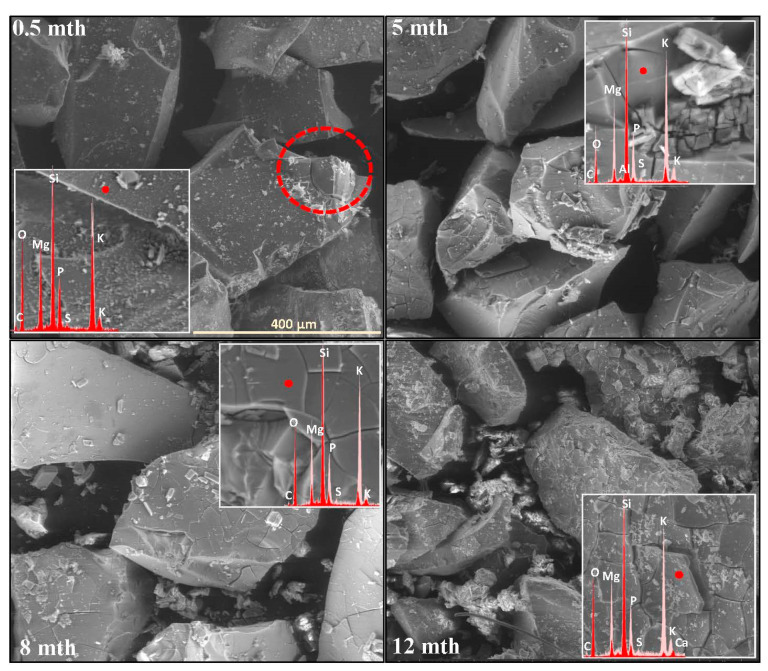
Micrographs illustrating surface changes in the 3S_6PM glass after 0.5, 5, 8, and 12 months of incubation, accompanied by EDS spectra from selected surface points (red spectrum) compared with the spectrum of the unaltered glass (light-pink spectrum). The main microphotographs were taken at 350× magnification, while the inset images were captured at 5000×. The scale bar shown in the first image (400 µm) applies to all other images in the figure.

**Figure 3:**
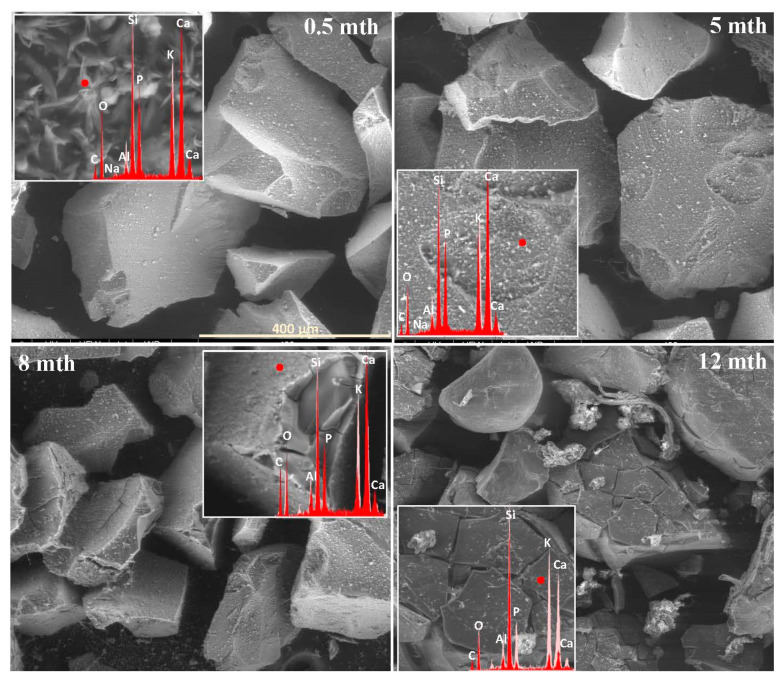
Micrographs illustrating surface changes in the 0S_6PC glass after 0.5, 5, 8, and 12 months of incubation, accompanied by EDS spectra from selected surface points (red spectrum) compared with the spectrum of the unaltered glass (light-pink spectrum). The main microphotographs were taken at 350× magnification, while the inset images were captured at 5000×. The scale bar shown in the first image (400 μm) applies to all other images in the figure.

**Figure 4:**
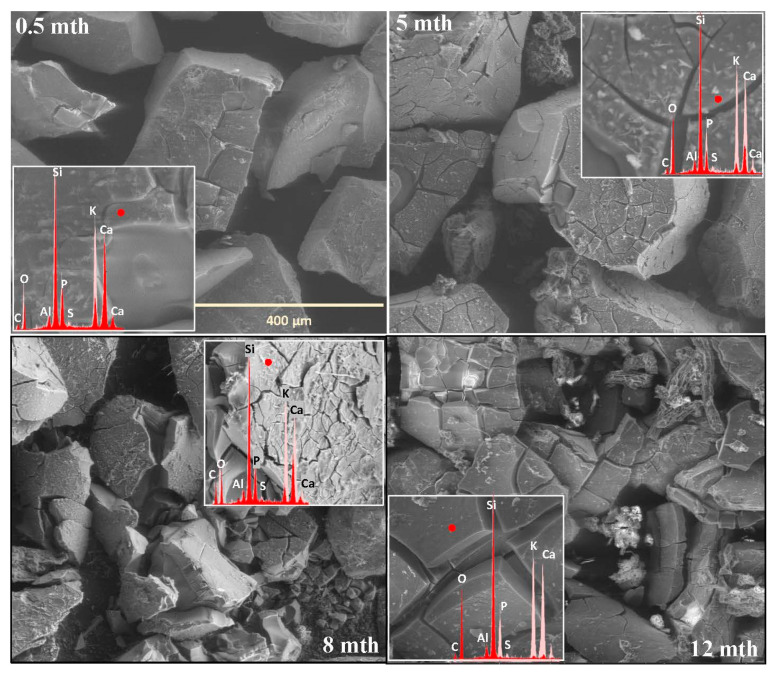
Micrographs illustrating surface changes in the 3S_6PC glass after 0.5, 5, 8, and 12 months of incubation, accompanied by EDS spectra from selected surface points (red spectrum) compared with the spectrum of the unaltered glass (light-pink spectrum). The main microphotographs were taken at 350× magnification, while the inset images were captured at 5000×. The scale bar shown in the first image (400 μm) applies to all other images in the figure.

**Figure 5:**
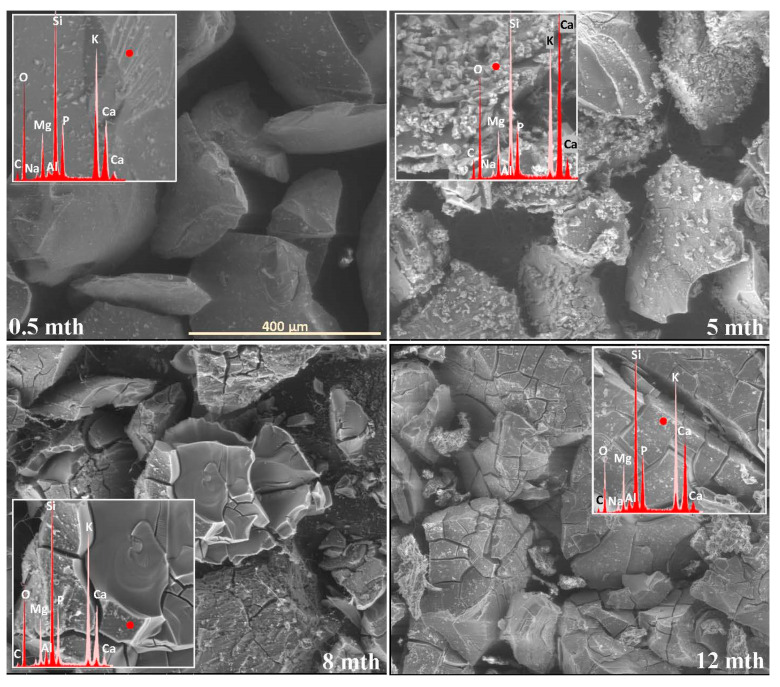
Micrographs illustrating surface changes in the 0S_6PMC glass after 0.5, 5, 8, and 12 months of incubation, accompanied by EDS spectra from selected surface points (red spectrum) compared with the spectrum of the unaltered glass (light-pink spectrum). The main microphotographs were taken at 350× magnification, while the inset images were captured at 5000×. The scale bar shown in the first image (400 µm) applies to all other images in the figure.

**Figure 6:**
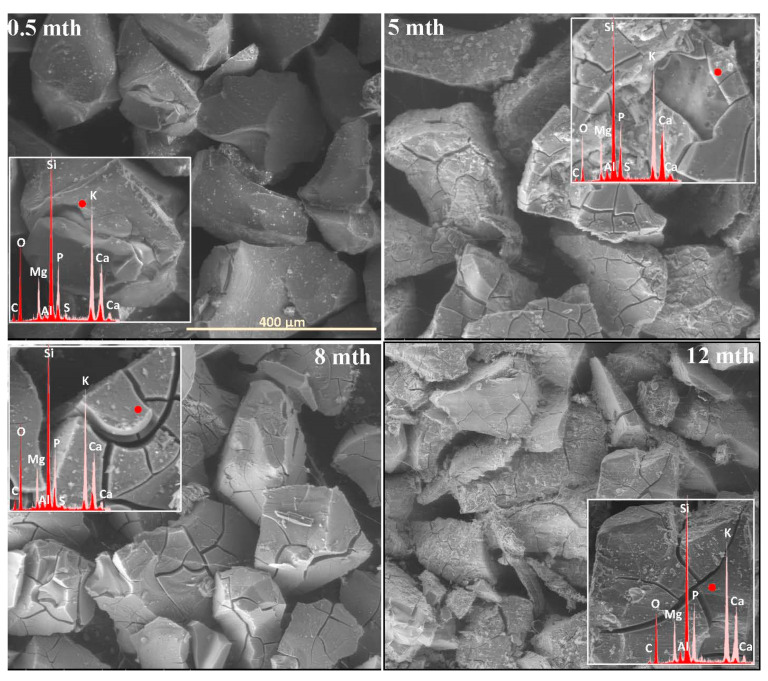
Micrographs illustrating surface changes in the 3S_6PMC glass after 0.5, 5, 8, and 12 months of incubation, accompanied by EDS spectra from selected surface points (red spectrum) compared with the spectrum of the unaltered glass (light-pink spectrum). The main microphotographs were taken at 350× magnification, while the inset images were captured at 5000×. The scale bar shown in the first image (400 µm) applies to all other images in the figure.

**Figure 7:**
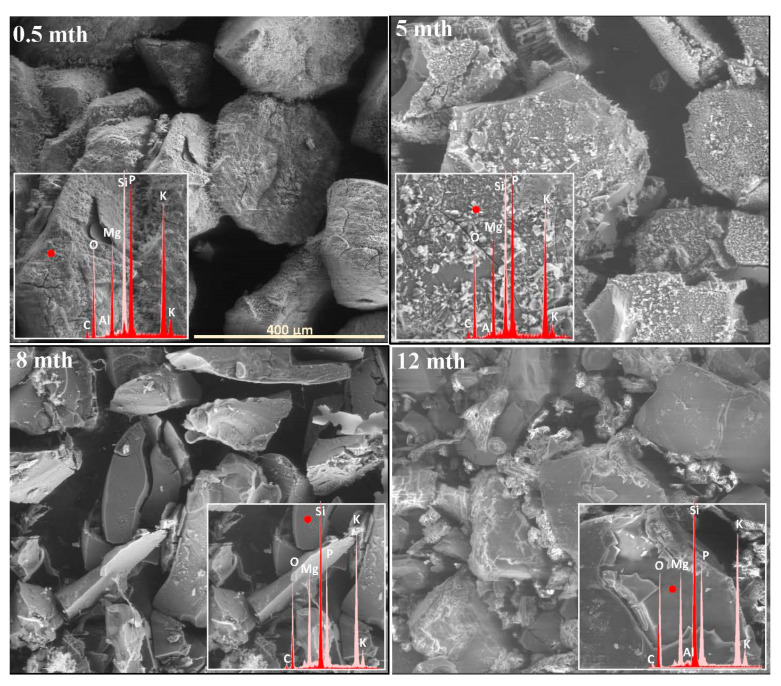
Micrographs illustrating surface changes in the 0S_10PM glass after 0.5, 5, 8, and 12 months of incubation, accompanied by EDS spectra from selected surface points (red spectrum) compared with the spectrum of the unaltered glass (light-pink spectrum). The main microphotographs were taken at 350× magnification, while the inset images were captured at 5000×. The scale bar shown in the first image (400 μm) applies to all other images in the figure.

**Figure 8:**
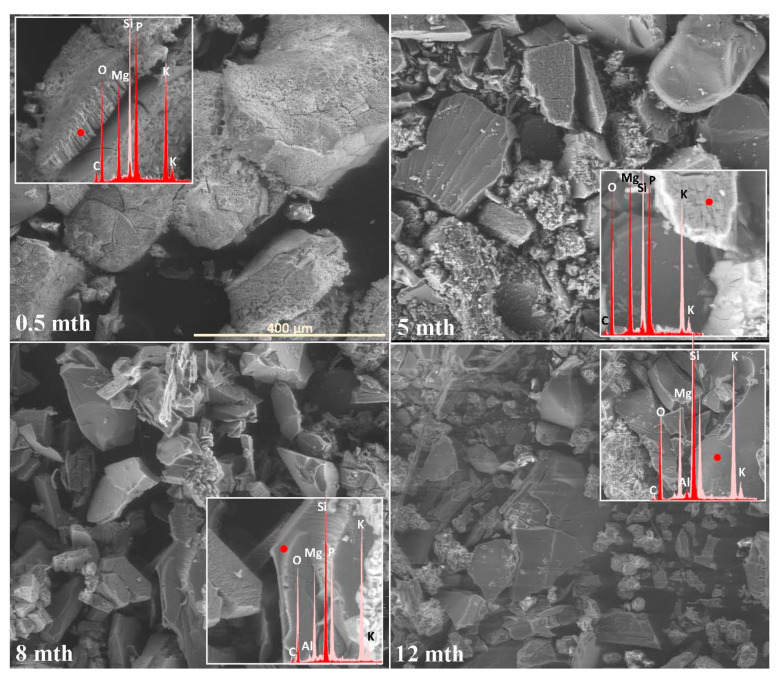
Micrographs illustrating surface changes in the 3S_10PM glass after 0.5, 5, 8, and 12 months of incubation, accompanied by EDS spectra from selected surface points (red spectrum) compared with the spectrum of the unaltered glass (light-pink spectrum). The main microphotographs were taken at 350× magnification, while the inset images were captured at 5000×. The scale bar shown in the first image (400 μm) applies to all other images in the figure.

The extent and scale of the changes occurring on the surfaces of the incubated glasses in contact with the soil solution were evaluated by examining the surface layer cracking, identifying the presence and characteristics of secondary precipitated phases, and analyzing the ion leaching intensity from superficial glass regions using EDS data. The collected data are presented descriptively in Table 1, accompanied by micrographs (Figure 1, Figure 2, Figure 3, Figure 4, Figure 5, Figure 6, Figure 7 and Figure 8) depicting incubated samples from all the studied compositions modified with 0 and 3 mol% sulfate units, after 2 weeks, as well as at 5, 8, and 12 months of incubation.

For glasses representing the XS_6PM system, the initial incubation period (first two months) under simulated spring conditions was marked by the intense crystallization of secondary products in the form of small (~5 µm) magnesium phosphate crystallites, which formed a discontinuous layer covering the entire surface of the granules. Nevertheless, while precipitates were already observed on the surface of the baseline sample after just two weeks (Figure 1: 0.5 mth), in the sulfate-bearing counterpart, the first signs of interaction with the soil solution appeared as localized cracks in the most weathering-prone areas (marked with a dashed line). Similar secondary phases were only observed from the second month onward, persisting until the end of the experiment (Table 1). The leaching of mobile components occurred at a low intensity and remained localized within the cracked regions (Figure 2). These processes continued in a similar manner throughout the months simulating spring conditions.

It is only with the temperature increase imposed by the simulated summer conditions (starting from the fourth month) that a distinct intensification of surface processes was observed. The entire outer layer of glass exposed to the soil medium developed a network of cracked, Si-enriched scales with only residual amounts of mobile ions, indicating an enhancement of ion exchange processes with the contact solution. This leaching intensification was noticeable from the fifth month in the baseline sample and from the third month in the sulfate-doped variant (Table 1; Figure 1 and Figure 2: 5 mth). Precipitation processes also became significantly more pronounced, as evidenced by the presence of large (40–50 μm), well-defined characteristic crystals composed of small subunits with visible gaps between them, of a chemical composition corresponding to magnesium phosphate (Figure 2: 5 mth: EDS spectrum). Additionally, on the surface of the baseline glass, distinct etch pits (marked with red circles) suggest the localized dissolution of the Si-O-Si glass network.

As the incubation progressed and temperatures dropped during the simulated autumn (after the seventh month), the Si-rich crust, initially forming as cracked scales, began to visibly disintegrate, exposing deeper layers of the material to direct contact with the soil solution and subsequently shifting the reaction front further into the glass structure (Figure 1 and Figure 2: 8 mth). Interestingly, the large precipitated magnesium phosphate crystals also responded to the temperature shift, undergoing mechanical decohesion—an effect particularly pronounced in the sulfate-doped glass.

The further temperature drop associated with the transition to winter-like simulated conditions, along with daily fluctuations around 0 °C, appeared to accelerate the fragmentation not only of the glass surface but also of entire sample fragments (Figure 1 and Figure 2: 12 mth).

Analogous to the MgO-containing system, the XS_6PC series also exhibited high reactivity in the acidic soil environment. However, while the baseline glass primarily developed a network of small, star-shaped precipitates (~5 µm in diameter), whose composition suggests the formation of calcium phosphate (Figure 3: 0.5 mth), the 3S_6PC sample exhibited additional features. In this case, besides localized precipitations of a similar nature, the surface also developed flaking cracks, indicating the localized release of mobile glass components (K^+^) into the contact solution (Figure 4: 0.5 mth).

As in the previous case, the temperature increase associated with simulated summer conditions appeared to intensify the processes at the glass–soil interface. For the base glass, this effect was largely limited to a slight enhancement in the surface cracking and further calcium phosphate crystallization, forming a discontinuous but dense network of secondary products covering the incubated fragments. In contrast, the 3S_6PC sample underwent extensive fracturing of the still-phosphate-coated surface, accompanied by a significant intensification of the leaching process, which began as early as the fourth month of incubation. In the baseline glass, in contrast, this effect was delayed, becoming noticeable only after six months (Figure 3 and Figure 4: 5 mth; Table 1).

The temperature drop during the transition from summer to autumn further accelerated the fragmentation of the altered layers. Nonetheless, in the sulfate-free sample, this phenomenon remained largely confined to areas most susceptible to weathering, such as edges and granules with highly developed surfaces. In contrast, the [SO_4_^2−^]-modified glass underwent extensive disintegration, affecting not only the surface layer but also entire fragments of the material (Figure 3 and Figure 4: 8 mth). Despite these transformations, the superficial regions of incubated granules in both cases remained coated with a discontinuous layer of calcium phosphates.

With the onset of simulated winter conditions, the mechanical degradation intensified, though the effect was significantly more pronounced in the sulfate-containing sample (Figure 3 and Figure 4: 12 mth). Notably, during this period of increased surface degradation, the previously observed phosphate deposits were no longer present, and only localized precipitates could be detected, which may suggest either the gradual dissolution of these secondary phases in the acidic peat environment or their mechanical decohesion.

Meanwhile, the baseline sample representing the mixed-alkaline-earth system appeared to exhibit the least interaction with the soil solution among all the tested compositions after two weeks of incubation, as no visible cracks or precipitates were observed on its surface (Figure 5: 0.5 mth). In contrast, its sulfate-modified counterpart showed localized fractures, characterized by the leaching of the most mobile glass components (Figure 6: 0.5 mth). Remarkedly, as the incubation progressed under simulated spring conditions, the presence of the most diverse secondary phases among all the tested systems was observed, reflecting the intricate interactions between the glass matrix constituents in the complex composition of the XS_6PMC system. Although both glass types developed magnesium phosphate and calcium carbonate phases, the baseline sample also exhibited calcium phosphate precipitates, while the sulfate-doped one formed double magnesium–potassium phosphates (Table 1). Notably, these secondary phases initially appeared as localized deposits, densely covering the surfaces of the 0S_6PMC and 3S_6PMC glasses between the 4th and 6th and 2nd and 4th months of incubation, respectively.

For the base glass, an increase in temperature promoted crystallization, accompanied by progressive surface fracturing (Figure 5: 5 mth). Conversely, in the sulfate-doped sample, summer-like conditions primarily drove mechanical surface degradation, leading to the intensified leaching of the mobile glass constituents into the surrounding solution (Figure 6: 5 mth, Table 1). The transition to autumn- and, subsequently, winter-like conditions significantly impacted the cohesion of the superficial glass layers, exacerbating both fracturing and mechanical disintegration. This process exposed the deeper, still-intact glass regions to the action of the soil solution (Figure 5 and Figure 6: 8,12 mth). During these simulated seasons, secondary phase precipitation was observed only sporadically, and predominantly in the form of calcium phosphate (with a single instance of calcium carbonate precipitation observed in the baseline sample) (Table 1).

Clearly, samples from the 10P series exhibited the highest reactivity in the soil environment, as evidenced by the pronounced surface cracking and the formation of a thick (~15 µm) needle-like layer of double magnesium–potassium phosphates from the earliest observation stage (Figure 7 and Figure 8: 0.5 mth).

The transition to summer-like conditions resulted in the gradual disappearance of this dense precipitate layer, occurring after the 6th and 5th months for the 0S and 3S_10M samples, respectively (Table 1). In both cases, this phase coincided with significant fragmentation of the incubated granules, indicating that the surface layer decohesion was primarily mechanical, though partial dissolution in the acidic peat environment cannot be excluded (Figure 7 and Figure 8: 5 mth). From the 5th month onward, a notable shift in the precipitation pattern of the sulfate-modified sample was observed, with the emergence of large, well-defined magnesium phosphate crystals, whereas the base glass no longer exhibited a tendency towards further crystallization. At this stage, both samples also showed a marked intensification of mobile ion leaching (Table 1). As the incubation progressed and the temperatures continued to drop, the fragmentation of the incubated material was further exacerbated, along with the mechanical decohesion of the precipitated phases—an effect particularly pronounced in the 3S_10PM sample (Figure 7 and Figure 8: 8,12 mth).

The data presented in Table 1 reveal a strong correlation between the degree of surface layer disintegration and both the intensity of the mobile ion leaching into the surrounding solution and the decohesion or thinning of the secondary precipitated phases. In all the studied systems, these effects manifested at earlier incubation stages and with greater intensity in the 3S samples compared to their baseline glass counterparts (Table 1). Consequently, the collected data suggest that sulfate species indirectly influence glass–soil solution interactions by enhancing the material’s susceptibility to fracturing and subsequently promoting the decohesion of the altered superficial layers during incubation.

This observation aligns with the DSC and MAS-NMR results discussed in the authors’ preceding study on the same materials and pointing to a reduction in thermal stability and an increase in network connectivity with increasing sulfur contents. Such structural modifications translate into the enhanced rigidity of the silicon–oxygen backbone, resulting, in turn, in a greater propensity for the surface cracking and exfoliation of the incubated specimens.

In the XS_6PM system, this effect was initially less pronounced between the 0S and 3S glasses but became evident in the later experiment stages—likely due to the already relatively high polymerization degree of the baseline glass (and its associated structural stresses) compared to other compositions within the [6P] series. Conversely, in the XS_6PC and XS_6PMC systems, the intensified fracturing of the 3S specimens was apparent throughout all the incubation stages. While numerous studies have reported that CaO-rich glasses exhibit lower susceptibility to cracking [47,48], attributed to the greater flexibility of the Si-O-Si framework when modified by calcium oxide compared to MgO [49], the introduction of highly covalent sulfate species occupying matrix voids reduces this initial elasticity. As a result, the network becomes more rigid, mirrored by the registered intensified surface fracturing. Glasses from the 10P system, which exhibited the highest degree of polymerization and the lowest thermal stability, appeared to be the most reactive, as from the earliest stages of incubation, the XS_10P samples displayed extensive fracturing across their entire surfaces. Nevertheless, even in this highly active system, the [SO_4_^2−^] addition further accelerated the interfacial degradation by promoting glass granule decohesion; however, similar to the XS_6PM system, this effect became most pronounced during the later incubation stages.

### 2.2. Sequence of Glass Component Release into Soil Solution

**Figure 9:**
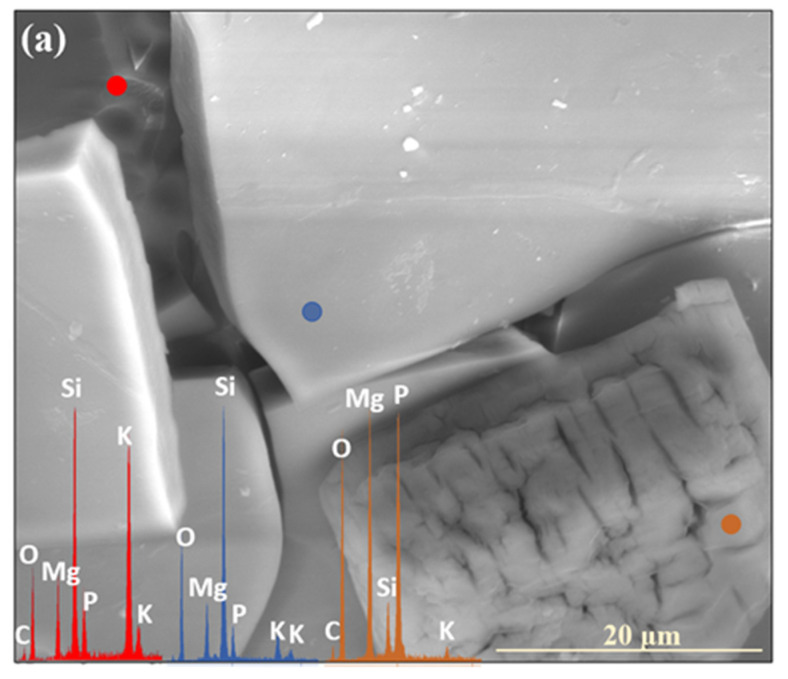

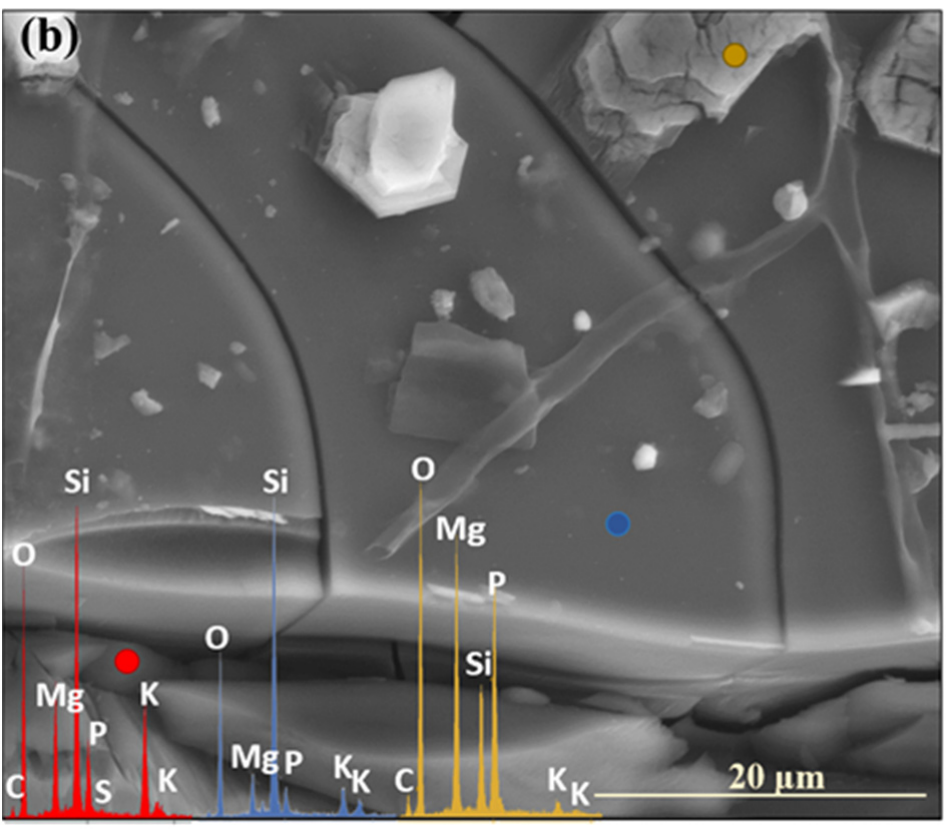
Micrographs and EDS spectra of selected surface points on 0S_6PM (**a**) and 3S_6PM (**b**) glasses after 8 months of incubation, illustrating the sequences of element release from the studied samples. The microphotographs were taken at 5000× magnification.

The release of glass matrix components from the XS_6PM system began with alkali ions, as evidenced in Figure 9a, where newly exposed—via fracturing—surfaces exhibit significantly increased K^+^ contents. This phenomenon is indicative of the preferential partitioning of this particular element in the process of ion exchange, making it the first to migrate toward near-surface regions (Figure 9a: red spectrum). Subsequently, albeit at a slower rate compared to K^+^, Mg^2+^ and PO_4_^3−^ ions were released, leaving behind a Si-enriched hydrated layer (Figure 9a: blue spectrum), which, in further stages, may either lose moisture during dry periods, leading to mechanical decohesion, or retain hydration and transition into a silica gel phase, which is progressively depleted of all mobile glass constituents (Figure 9a: yellow spectrum).

Similar to the base composition, the sulfate-modified glass also exhibited the preferential release of potassium ions, as already after two weeks of incubation, the SEM-EDS analysis of the selected surface region of the 3S_6M sample revealed a slight depletion of K^+^ from the glass matrix, while the concentrations of the other major glass components remained unchanged (Figure 9b).

Further interpretation of the SEM-EDS data suggests that sulfate ions were the next species released into the soil solution. The region exposed following the disintegration of the altered layer was characterized by a reduced concentration of both K^+^ and SO_4_^2−^, while the levels of other mobile species, such as Mg^2+^ and PO_4_^3−^, remained unchanged (Figure 9a: red spectrum). The reduced leaching tendency of Mg^2+^ and PO_4_^3−^ was likely due to the rapid attainment of supersaturation in the thin moisture film adhering to the glass surface, thereby diminishing the reaction affinity. Apparently, the subsequent precipitation of magnesium phosphates (Figure 9a: yellow spectrum) depleted these ions from the contact solution, restoring the leaching potential, as evidenced by the near-complete depletion of mobile glass constituents in the Si-rich altered layer (Figure 9a: blue spectrum).

Analogous to the MgO-containing system, the XS_6PC series also exhibited the preferential leaching of potassium ions into the contact solution. Observations conducted after two weeks of incubation revealed a slight depletion of K^+^ relative to its initial concentration, accompanied by the accumulation of Ca^2+^ ions in the superficial regions of the tested glasses (Figure 5: 0.5 mth). Notably, phosphate ions (PO_4_^3−^) also concentrated in the near-surface regions, eventually coprecipitating with Ca^2+^ to form a thin layer of calcium phosphates (Figure 5: 5 mth).

**Figure 10:**
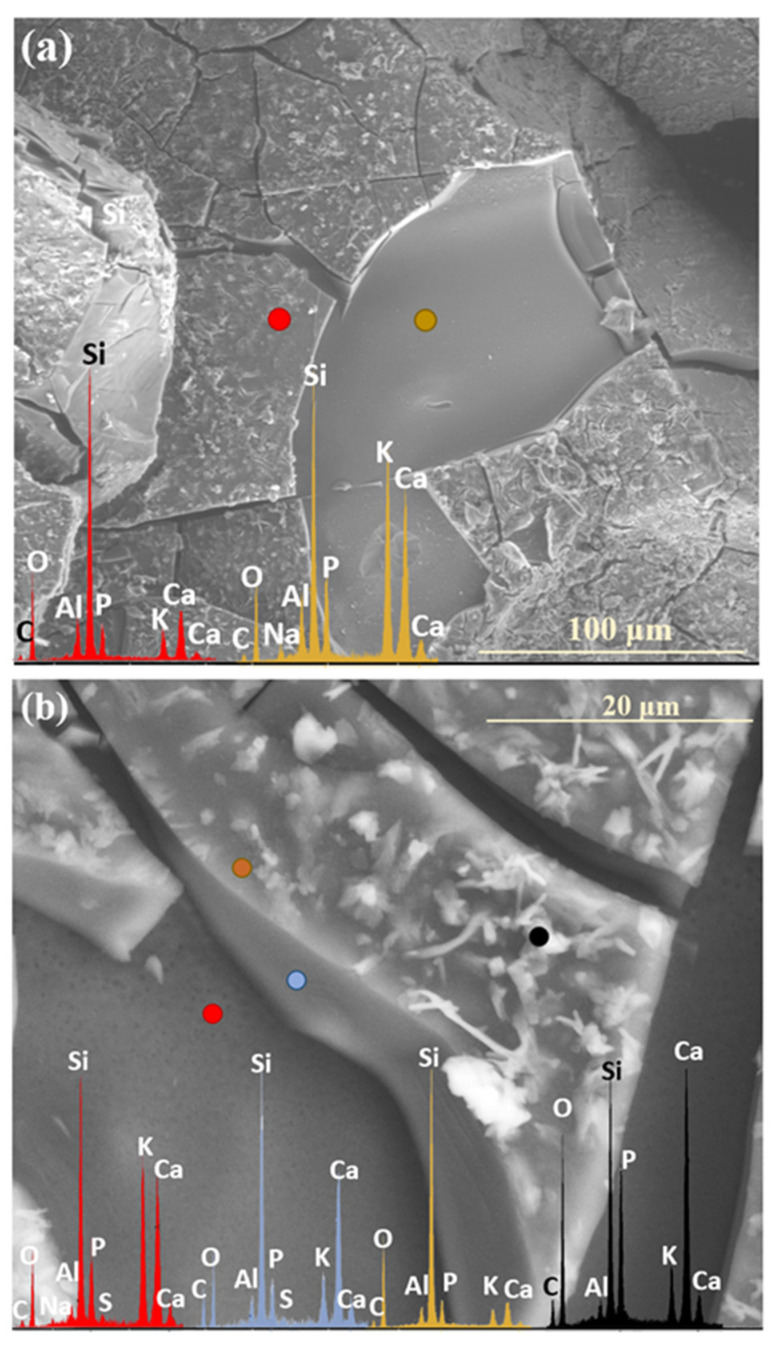
Micrographs and EDS spectra of selected surface points on 0S_6PC (**a**) and 3S_6PC (**b**) glasses after 7 and 5 months of incubation, respectively, illustrating the sequences of element release from the studied samples. The microphotographs were taken at 1000× (**a**) and 5000× (**b**) magnification.

Although a similar tendency for calcium retention within the hydrated Si-rich altered layer was previously reported by Chave et al. [50], who proposed the passivating effect of this phase, in the present study, no such diffusion barrier effect was observed, even when the entire glass surface was covered with a precipitate layer (Figure 5: 8 mth—diminished K^+^ concentration). This discrepancy is likely attributed to the discontinuous nature of the registered secondary product, which did not form a uniform protective coating, as well as to the pronounced propensity of the superficial glass regions to crack and exfoliate under cyclic wet–dry conditions, which facilitated the progression of the reaction front deeper into the pristine glass, ultimately accelerating the glass alteration process (Figure 10a).

When evaluating the preferential release of K^+^ over Ca^2+^, the mineralogical profile of the soil environment must also be considered. The significantly higher concentration of calcium ions in peat soil (Table 2) likely reduces the driving force for Ca^2+^ leaching, further emphasizing the complexity of soil–glass interactions and the importance of environmental variables in governing glass dissolution mechanisms.

SEM-EDS analysis of selected points on the surface of the 3S_6PC sample (Figure 10b) suggests that the sequential release of K^+^ and SO_4_^2−^ ions follows a pattern analogous to that observed in the 3S_6PM system. The sulfate ion concentration gradually decreases from the freshly exposed glass surface (red spectrum), through the altered layer (blue spectrum), and into the Si-enriched crust (yellow spectrum), highlighting the progressive modification of the outermost glass regions during incubation.

**Figure 11:**
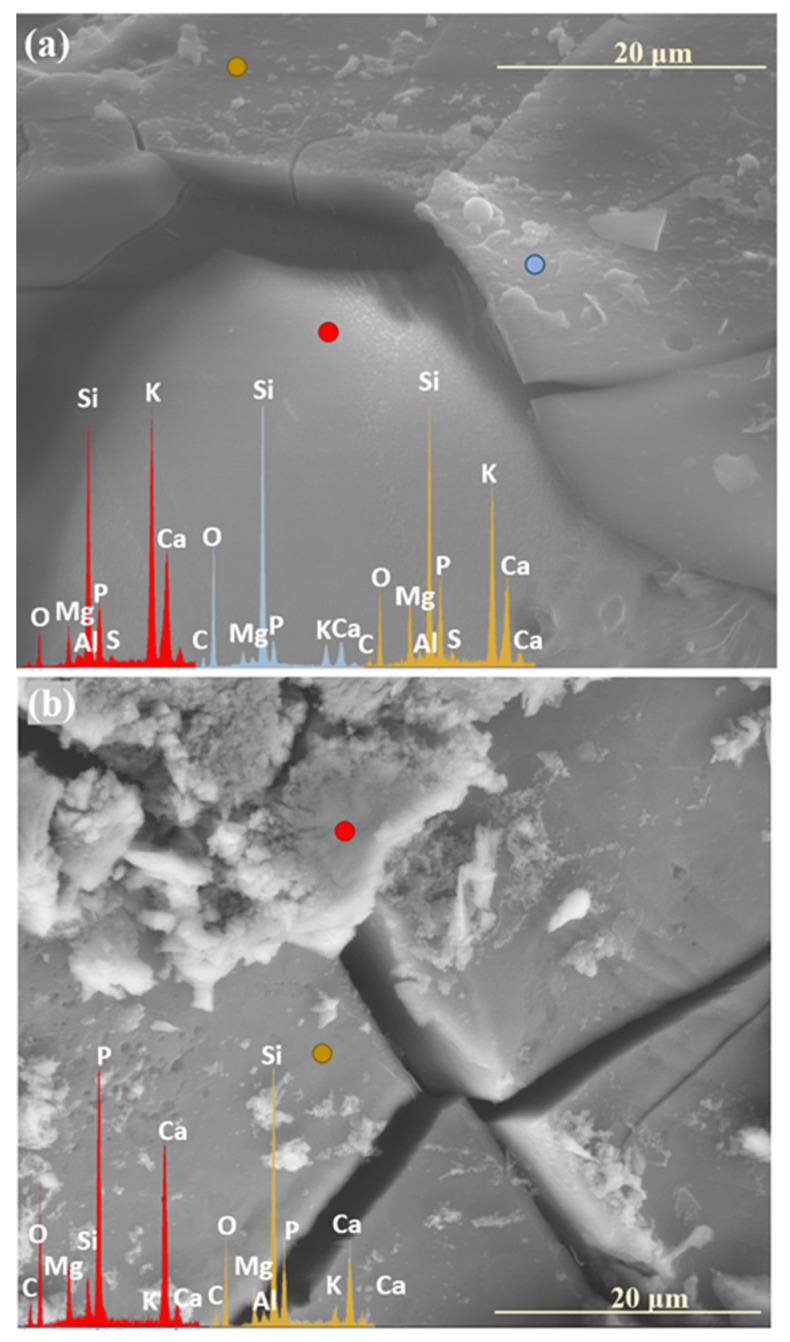
Micrographs and EDS spectra of selected surface points on 3S_6PMC (**a**) and 0S_6PMC (**b**) glasses after 0.5 and 7 months of incubation, respectively, illustrating the sequences of element release from the studied samples. The microphotographs were taken at 5000× magnification.

Similar to the previously discussed systems, the XS_6PMC series also exhibited the highest mobility of K⁺ ions, the increased concentration of which was detected in the near-surface regions, newly exposed due to the decohesion of the altered layer (Figure 11a: red spectrum). This observation further supports the preferential involvement of K^+^ in interdiffusion processes, leading to its early transport toward the glass surface as the first matrix element to be released. In contrast, the edge region of the resulting cavity is predominantly silica-enriched, containing only residual amounts of other mobile ions (K^+^, Mg^2+^, Ca^2+^, PO_4_^3−^) (blue spectrum).

Analogous SEM-EDS analyses conducted on the base glass of this system revealed a trend similar to that observed in the XS_6PC samples, marked by the accumulation of Ca^2+^ and [PO_4_^3−^] ions in the near-surface regions (Figure 11b: yellow spectrum). This behavior is attributed to the rapid attainment of supersaturation in the contact solution with respect to these components, promoting their precipitation as secondary phases (red spectrum).

**Figure 12:**
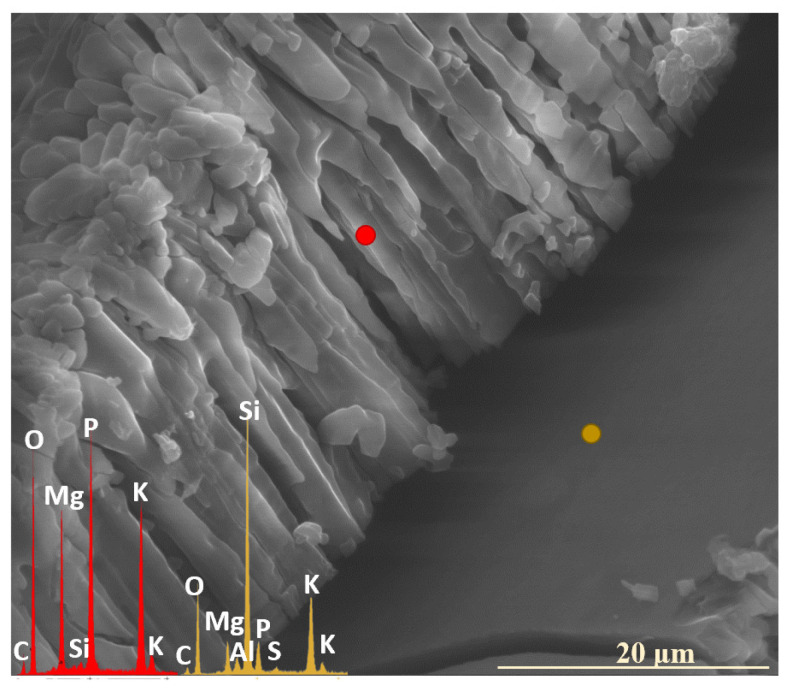
Micrograph and EDS spectra of selected surface points on 3S_10PM glass after 0.5 month of incubation, illustrating the sequence of element release from the studied sample. The microphotograph was taken at 5000× magnification.

**Figure 13:**
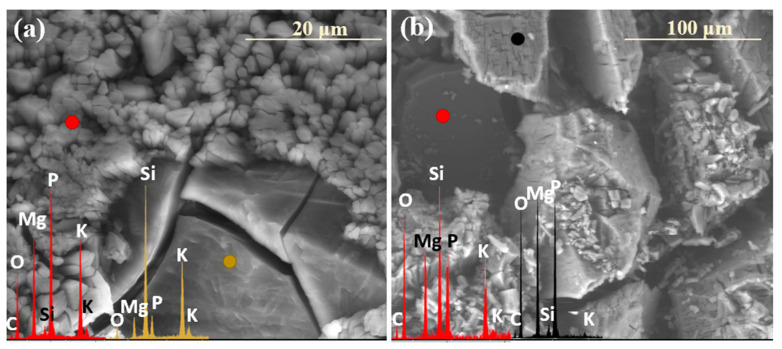
Micrographs and EDS spectra of selected surface points on 3S_10PM glass after 3 months (**a**) and 5 months (**b**) of incubation, illustrating the retention of ions within the hydrated silica layer.

Interestingly, in the 10P system, which exhibited the highest overall reactivity, no preferential release of any specific mobile glass constituents into the soil solution was observed, and all the major ions (K^+^, Mg^2+^, PO_4_^3−^, and SO_4_^2−^) appeared to leach simultaneously instead (Figure 12: yellow spectrum). Notably, the retention of these ions within the hydrated silica layer varied depending on the type of secondary crystalline phase that formed at different incubation stages. In the early phase of the experiment, when double magnesium–potassium phosphates precipitated, K^+^ was preferentially retained within the altered zone (Figure 13a), while during the later months, which favored the crystallization of magnesium phosphate, Mg^2+^ and PO_4_^3−^ accumulated within the altered layer (Figure 13b).

**Figure 14:**
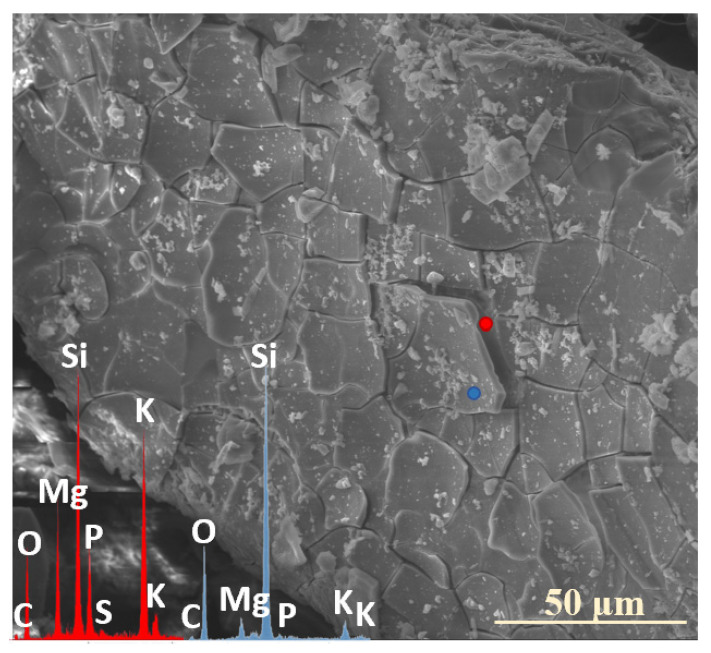
Micrograph of the 3S_6PM glass surface after 12 months of incubation, showing the presence of SO_4_^2−^ species on the freshly exposed glass surface. The microphotograph was taken at 5000× magnification.

Remarkedly, SO_4_^2−^ species were predominantly released during the initial incubation stages, likely due to their high leachability under acidic conditions [51,52]. In all glasses representing the [6P] series, sulfur was detected in the EDS spectra only up to the 2nd month of incubation (or up to the 3rd month in the XS_6PC system), whereas at later stages, it appeared only sporadically, primarily in freshly exposed areas following surface exfoliation (Figure 14). In contrast, in the highly reactive XS_10PM system, this element was detected only in the first two weeks, confirming the most rapid leaching dynamics of the higher P_2_O_5_ composition among all the studied compositions (Figure 12: yellow spectrum).

Given that, according to the observations carried out, K⁺ leaches preferentially, followed by alkaline-earth cations (Mg^2+^, Ca^2+^) and PO_4_^3−^ and SO_4_^2−^ species, while the silicon–oxygen framework remains largely intact, the registered elemental release patterns suggest a selective, incongruent dissolution process. This acidic pH-driven, selective removal of mobile glass components leads to the rapid saturation of the limited-contact soil solution with the leached ions. As a result, glass constituents exceeding their solubility limit in the surrounding medium precipitate onto the outermost surface regions, forming a layer of secondary minerals.

### 2.3. Characterization of Precipitation Layers

**Figure 15:**
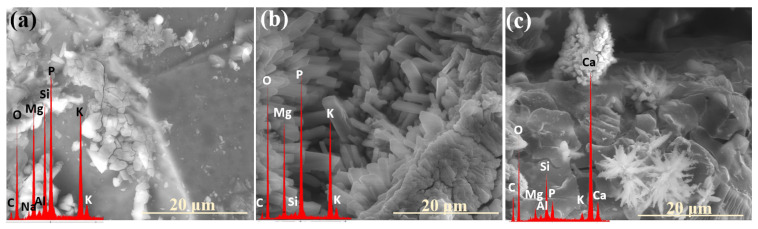
Exemplary secondary phases detected during the glass incubation experiment: double magnesium–potassium phosphates on the surfaces of 0S_6PM (**a**) and 0S_10PM (**b**) samples, as well as calcium carbonate observed on the 3S_6PMC glass (**c**). The microphotographs were taken at 5000× magnification.

Analyzing the data presented in Table 1, it becomes evident that the primary factor governing the formation of precipitating secondary phases is the chemical composition of the given glass. Accordingly, the presence of magnesium phosphates, as well as of double phosphates of potassium and magnesium in systems containing MgO as an alkaline-earth network modifier, is to be expected. Interestingly, for compositions with reduced P_2_O_5_ contents, the latter phase appeared only sporadically during the first month of the experiment (Figure 15a), whereas in the 10P series, it predominated, covering the entire superficial area of granules with a dense, thick (~16 µm) layer (Figure 15b).

Notably, while potassium struvite typically crystallizes into irregular tetrahedral shapes (disphenoids) when synthesized, the gel diffusion method tends to produce the characteristic needle-like morphology, analogous to the one observed in this study. It can therefore be inferred that the initial hydration of the surface layers of incubated glasses promotes the organization of leached ions (K^+^, Mg^2+^, PO_4_^3−^) into the double-phosphate structure detected. Additionally, as reported by Türk et al. [53], the coprecipitation of potassium to form struvite is favored under slightly alkaline conditions (pH < 8), whereas magnesium phosphate crystallizes at higher pH values. This observation underscores the critical role of the contact solution’s pH in governing the crystallizing phase and offers valuable insights into the sequence of secondary product formation [54,55].

During the early stages of the experiment, when the alkalization of the contact solution was minimal due to localized and less intense ion leaching, the formation of double phosphates was promoted. In contrast, at later stages, higher pH values in the thin moisture film adhering to the glass surfaces favored the precipitation of Mg_3_(PO_4_)_2_. Furthermore, samples from the [10P] system were found to alkalize the surrounding environment to a lesser extent than those with lower P_2_O_5_ contents (as evidenced by long-term pH monitoring, the results of which are discussed in the authors’ preceding study on the same materials, serving as a complementary investigation to this work), which explains the tendency of the XS_10PM glasses to preferentially form a potassium-containing struvite analogue, while magnesium phosphate appears in the XS_6PM system. Also worth emphasizing is the fact that not only the type of the crystal itself but also its morphology and habit are pH-dependent [53].

Based on the research of Prywer et al. [56], who studied struvite isomorphous phases, one may infer the conditions under which the observed Mg₃(PO₄)_2_ phase was formed. It has been established that at lower pH values, single, poorly defined crystals are generally observed (as seen in Figure 1: 0.5 mth), whereas an increase in the pH promotes the precipitation of distinct, porous forms exhibiting a unique morphology that gives the impression of a structure composed of smaller subunits (Figure 9a: yellow spectrum).

While both the above-discussed phosphates are known to form in alkaline conditions, calcium phosphate precipitates over a broad pH range; however, depending on the acid/base properties of the environment, it can adopt various sizes, morphologies, and degrees of crystallinity [57,58,59,60,61].

Remarkedly, the ‘desert-rose’ form exhibiting poor crystallinity, observed with varying intensity in the XS_6PC system throughout the experiment, is promoted when the solution is slightly acidic (pH ~ 5–6) [59]. Such a statement, in turn, would suggest that CaO-containing compositions induce only the mild alkalization of the surrounding environment; but, given that the Ca^2+^ ions tended to accumulate in the superficial regions of the glass (Figure 10b: black spectrum) and therefore did not contribute to the formation of Ca^2+^(OH^−^)_2_ species in the contact solution, this conclusion appears well founded.

In contrast, in the XS_6PMC system, which is more prone to alkalizing the contact solution due to the additional presence of Mg^2^⁺ cations, another poorly crystalline form of calcium phosphate was observed: agglomerates of cloudlike, shapeless granules, known to precipitate at higher pH values of ~7.5 [57] (Figure 11b: red spectrum). Interestingly, in this system, the dendrite-like calcium carbonate formations were also locally observed (Figure 15c), with their presence indicating the involvement of carbonate ions in the contact solution (from either biotic or atmospheric sources) and a mildly alkaline environment (pH ~ 8–9) [60,61]. It is important to highlight that in this most complex composition, all the aforementioned phases were observed, reflecting the multicomponent nature of the system and the intricate relationships between the individual components of the glass matrix, as well as their diverse local effects on the pH of the contact solution.

It was observed that in none of the cases did the precipitated layer of secondary minerals establish a complete physical barrier to ion exchange processes. In the [6P]-series compositions, crystallization products settled on the altered glass surfaces in a fragmented, discontinuous manner, rather than forming an impermeable shield (Figure 3: 5 mth). Conversely, in the higher-P_2_O_5_ compositions, the precipitation layer exhibited the potential to act as a diffusion barrier; however, the presence of extensive cracking disrupted its integrity, enabling water penetration and consequently facilitating the transport of exogenous protonated species toward the pristine glass (Figure 12).

**Figure 16:**
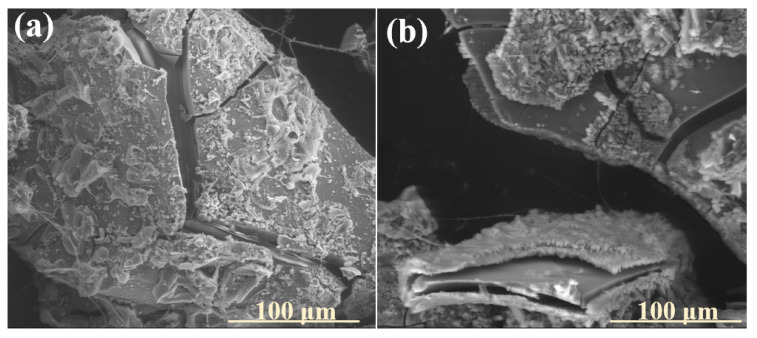
Micrographs of the surfaces of 3S_6PMC (**a**) and 3S_10PM (**b**) glasses after 6 months and 4 months of incubation, respectively, showing the tendency for the decohesion or mechanical disintegration of the precipitation layers. The microphotographs were taken at 1000× magnification.

Furthermore, in both cases, the precipitates demonstrated a pronounced tendency toward decohesion or mechanical disintegration, particularly under temperature fluctuations associated with seasonal transitions from warmer to colder periods (Figure 16a). Additionally, the structurally distinct nature of the precipitated layer, relative to the outermost altered glass surface, likely introduced additional stress, further exacerbating the cracking and mechanical separation (Figure 16b).

Notably, sulfate species were found to play no role in the formation of secondary phases accumulating on the glass surface. Due to their relatively low concentration compared to other glass constituents, sulfate ions do not oversaturate the contact solution but instead migrate away from the glass surface and diffuse into the surrounding soil substrate.

### 2.4. Mechanism of Glass–Soil Interaction

Given the above considerations, the observed interaction between glass and the soil substrate appears to occupy an intermediate position between two well-documented glass deterioration processes: degradation in liquid media (where rapid dealkalization occurs through ion exchange with exogenous positively charged species from the surrounding solution, and the released glass components are promptly removed from the material’s immediate vicinity—limiting oversaturation of the adjacent solution and, consequently, secondary phase precipitation), and alteration under atmospheric conditions (e.g., RH < 100%, where instead of being leached into a bulk liquid, glass constituents are redistributed within the hydrated alteration layer, modifying its composition and porosity differently, compared to aqueous environments). Conversely, in the temperate mid-European climate, such as that of Poland, the soil moisture conditions fluctuate on short time scales, influenced by weather patterns and seasonal changes. As a result, potential vitreous fertilizers may experience discontinuous exposure to aqueous media, undergoing alternating cycles of wet and dry conditions of varying durations, creating a dynamic and non-static corrosion environment.

**Figure 17:**
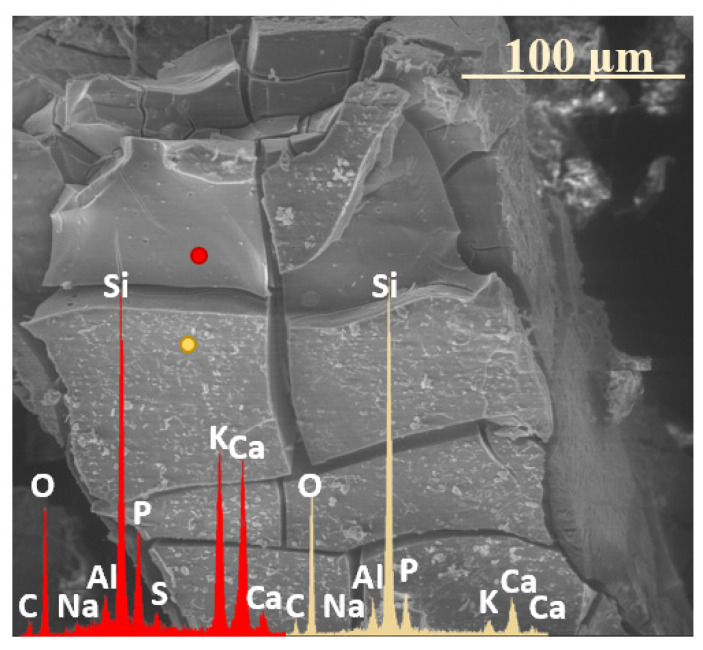
Micrograph illustrating the progression of the reaction front into the bulk glass through the decohesion of alteration layers, based on the observations of the 3S_6PC glass after 11 months of incubation. The microphotograph was taken at 1000× magnification.

Accordingly, the conditions imposed on the samples during the ‘in vivo’ experiment were designed to replicate this periodic variability, allowing for a more accurate prediction of their behavior in real-field applications. This fluctuation in the environmental conditions explains the layered structure observed in the near-surface regions of the incubated glasses throughout the experiment: periods of increased water availability promoted interdiffusion processes, facilitating the penetration of hydrogenated species from the solution into the glass while simultaneously driving the outward migration of mobile glass constituents. This dynamic exchange leads to the formation of diffusion (alteration) layers, akin to those observed in glass corrosion under liquid immersion. Following this stage of heightened water supply, subsequent dry periods induce the progressive dehydration of the altered zone, weakening the superficial glass regions and rendering them preferential planes along which the decohesion of the alteration layers occurs (Figure 17). Beyond periodic soil wetting episodes, this mechanical separation of glass fragments was particularly evident immediately after temperature drops associated with the transition from summer to autumn, highlighting the differing thermal expansion coefficients of the bulk glass and the weathered layer. While numerous studies have reported the diffusion-blocking function of the outermost altered glass layer [7,18,62,63], the present experiment suggests a contrasting behavior: the brittle, Si-enriched superficial zone—prone to fracturing both parallel and perpendicular to the glass surface—created a unique network of pathways that facilitated water penetration toward the pristine glass. However, the most impactful factor appeared to be the scaling phenomenon itself, which directly accelerated glass–soil interactions by exposing fresh, unaltered surfaces to the corrosive effects of the soil solution (Figure 17).

It is postulated that these alternating episodes of wetting and drying, coupled with cyclic temperature fluctuations (on both the diurnal and seasonal scales), establish a self-reinforcing feedback loop. As surface scaling advances the alteration front deeper into the pristine glass, the increased exposure to soil moisture enhances ion leaching, subsequently leading to the formation of a hydrated silica crust. During dry periods, this crust contracts and readily disintegrates, perpetuating the cycle. This hypothesis is further supported by the observed correlation between the extent of the surface fragmentation and the quantity of the leached mobile glass elements (Table 1).

The conditions employed in the conducted experiment—namely, the high surface-area-to-volume (SA/V) ratio, indicative of solution-dominated conditions, and the closed-system character of the incubation—exerted a significant influence on the processes occurring at the glass surfaces. Due to the acidic nature of the soil substrate (peat) and the resulting abundance of hydrogenated species (H^+^, H_3_O^+^) in the contact solution, the predominant alteration mechanism involved interdiffusion, leading to the progressive hydration of the superficial material layers, as described by Equations (1) and (2), illustrating the predominant reactions involved in the glass corrosion process [25,64]:

(1) $$\equiv Si-O^{-}-M_{\left(glass\right)}^{+}+H_{\left(sol.\right)}^{+}\to \equiv Si-{OH}_{\left(glass\right)}+M_{\left(sol.\right)}^{+}\left(\equiv represents\;the\;silica\;bonds\right)$$

(2) $$\equiv Si-O-M_{\left(glass\right)}^{+}+H_{3}O_{\left(sol.\right)}^{+}\to \equiv Si-{OH}_{\left(glass\right)}+M_{\left(sol.\right)}^{+}+H_{2}O$$

(3) $${\equiv Si-O-Si\equiv }_{\left(glass\right)}+{OH}_{\left(sol.\right)}^{-}⇌{\equiv Si-OH}_{\left(glass\right)}+\equiv Si-O_{\left(glass\right)}^{-}$$

(4) $${\equiv Si-OH}_{\left(glass\right)}+HO-{Si\equiv }_{\left(glass\right)}⇌{\equiv Si-O-Si\equiv }_{\left(glass\right)}+H_{2}O$$

Another direct consequence of reduced water availability was the rapid alkalization of the contact solution, driven by proton consumption and the subsequent formation of K^+^OH⁻ species (alternatively Mg^2+^2(OH^−^), with the less frequent appearance of Ca^2+^2(OH^−^) as Ca^2+^ ions, which were found to be retained in the near-surface glass regions). As-generated alkaline conditions lead to substantially more aggressive attacks directly affecting the silicate backbone via Si-O-Si bond hydrolysis (Equation (3)). Such visible marks of glass network dissolution resulting from the prolonged action of the alkaline medium in irregular surface areas constitute pits, occasionally detected on the tested material surfaces (see Figure 2: 8 mth, left upper corner).

**Figure 18:**
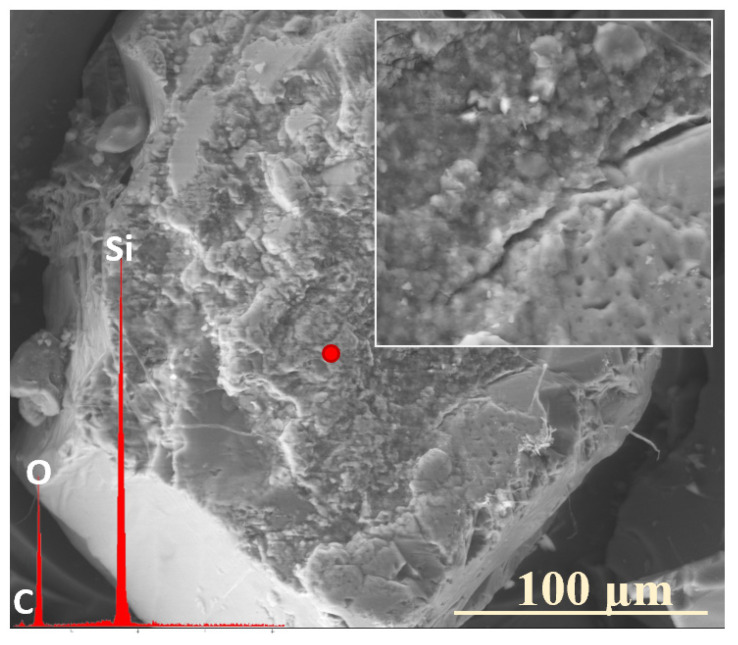
Silica gel layer observed on the surface of 3S_10PM glass after 10 months of incubation. The microphotograph was taken at 1000× magnification.

Despite the ion exchange process facilitating the hydration of the superficial glass region (Equation (4)), the formation of a distinct, gel-like, Si-enriched layer was only sporadically evidenced (Figure 18). It is believed that the periodic wet–dry cycles inhibited the stabilization of this alteration product—either due to water evaporation or the systematic exfoliation of the silica crust. Consequently, the occasional presence of silica gel was primarily observed under winter-like conditions, where the lack of air circulation (imposed by the limitations of the climate chamber) allowed for prolonged moisture retention.

Notably, the surface alteration processes recorded in this experiment exhibit substantial similarities to those well documented for glass deterioration in immersion conditions, as the analysis of the ‘in vivo’ experimental results confirms the occurrence of three key processes [18,19,65,66,67]: (1) the adsorption of a thin layer of moisture at the superficial glass area, (2) the proceeding of three parallel and interconnected reactions: ion exchange, hydration, and hydrolysis, as well as (3) the precipitation of the secondary product layer. However, the primary distinguishing factor between glass weathering in a soil medium versus a liquid environment is the water availability, which emerged as the critical factor governing the rate of glass alteration under the experimental conditions. In addition to the moisture dynamics, the glass composition is also known to play a role in controlling the reactions between glass constituents and environmental species, further influencing the overall alteration behavior.

**Figure 19:**
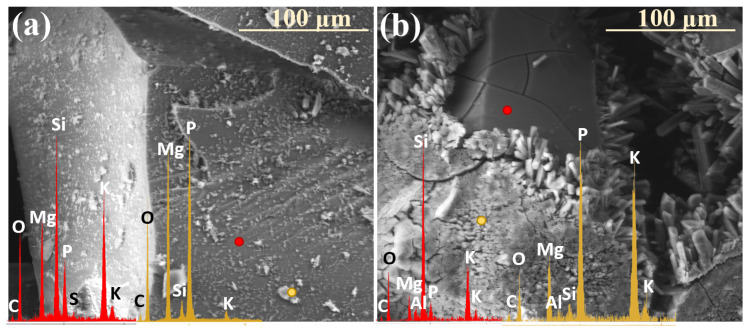
Comparison of the chemical activities of glasses with lower (**a**) and higher (**b**) P_2_O_5_ contents, based on 3S_6PM and 3S_10PM samples after 2 months of incubation. The microphotographs were taken at 1000× magnification.

In this study, however, only minor differences were observed in the behavior of the tested compositions in the soil environment, when comparing compositions belonging to the same series: 6P compositions. As discussed in Section 2.1, these differences primarily manifest in the material’s tendency to undergo fracturing, a process significantly intensified by the presence of sulfate units. Nonetheless, among the 6P compositions, the system that exhibited the lowest initial reactivity appears to be the XS_6PMC variant. This phenomenon can be attributed to the well-documented mixed-alkali-/-alkaline-earth effect, which increases the activation energy required for ion hopping between sites within the vitreous matrix, thereby hindering the ionic diffusion process [68,69]. Clearly, the P_2_O_5_ content plays a far greater role in controlling the glass reactivity than the specific type of alkaline-earth oxide modifier, as evidenced by the 10P system, which, despite having the highest network connectivity, was found to be the most chemically active. It is well known that introducing phosphorus pentoxide into silicate-based glass matrices enhances their hygroscopic properties while simultaneously reducing their resistance to humidity, as phosphate glasses are notorious for their rapid deterioration upon exposure to atmospheric moisture due to the presence of readily hydrated P-O-P bridges [14,15,68,70,71,72]. Therefore, it is assumed that the strong hydrophilic nature of [PO_4_^3−^] species renders these glasses highly susceptible to moisture-induced degradation; the more water molecules adsorbed at the surface, the deeper and faster the hydrolysis and hydration processes and the higher the chemical potential of the adsorbed ‘solution’, ultimately accelerating the leaching of mobile cations (Figure 19). Importantly, these considerations remain valid as long as phosphorus enters the vitreous matrix predominantly in the form of isolated $Q_{P}^{0}$ units, which, as indicated by previous research on similar materials, applies to glasses containing up to 15 mol% P_2_O_5_ [15,73,74]. Beyond this threshold, the structural role of phosphorus may undergo a significant shift, potentially reversing its effect on the overall glass reactivity, particularly in the presence of Fe as an introduced micronutrient [75,76].

Clearly, for glasses with the same P_2_O_5_ contents, variations in the type of network-modifying alkaline-earth oxide influence their interaction with the soil environment not only by affecting their propensity for fracturing but also by determining the nature of the crystallizing secondary phases and their further role in the glass–soil interface reactions. In the XS_6PM system, the precipitated magnesium phosphates appeared as large, well-defined crystals scattered across the glass surface, and their localized distribution did not present a significant barrier to ion exchange. Similarly, in the XS_6PMC system, the secondary phases also formed in a dispersed manner, and even during periods of increased precipitation, the resulting layer remained discontinuous. In contrast, calcium phosphates in the XS_6PC system initially formed a more distinct barrier, but only during the early stages of incubation, when the lack of cracks and the absence of surface mechanical degradation had not yet facilitated the advancement of the reaction front into the deeper glass matrix. Notably, in the 10P system, the precipitation layer appeared dense and uniform; however, the glass itself exhibited a high degree of fracturing, which enhanced the accessibility of the contact solution to the glass surface.

Importantly, it may be claimed that the existence of a superficial area of secondary phases is responsible for regulating the release of nutrients into the soil environment by virtue of the temporary immobilization of the elements within the composition of such a precipitation layer itself. It should be noted that all the observed phases display low solubility in water, preventing nutrient runoff, and are characterized by much higher acidic pH values in the environment [53,77,78,79].

From a practical standpoint, this precipitation-driven ion retention may be considered a natural strategy for controlled nutrient release, as the remobilization of useful ions is triggered under the influence of roots’/microorganisms’ acidic exudates. However, it must be stressed that calcium phosphates belonging to the apatite family exhibit the lowest solubility in acidic environments compared to other observed phases. This characteristic should be carefully considered when designing CaO-containing vitreous fertilizers, as the re-release of immobilized nutrients from these phases is expected to be the slowest [77].

### 2.5. Influence of Soil Environment

It is crucial to highlight the multitude and complexity of factors influencing the processes occurring at the soil–glass interface. Many of these factors, due to their inherently unpredictable nature, cannot always be fully accounted for when describing such interactions. Beyond the evident impact of seasonal or even daily weather fluctuations—which significantly affect the soil moisture levels, the concentrations of dissolved organic and inorganic compounds, and, consequently, parameters such as the electrical conductivity (EC) and redox potential (Eh)—the chemical and mineralogical properties of the soil itself play a pivotal role in shaping these processes.

A compelling confirmation of this influence is the observed effect of the glass composition on the ion mobility within the application environment. The acidic peat substrate used in the incubation experiments, characterized by a significant calcium ion content, may have aided in both the retention of these ions within the silica-enriched hydrated layer and the crystallization of secondary calcium phosphates. Conversely, the presence of exogenous carbonate ions—either biotically generated or derived from atmospheric CO_2_—promoted the formation of calcium carbonate precipitates.

**Figure 20:**
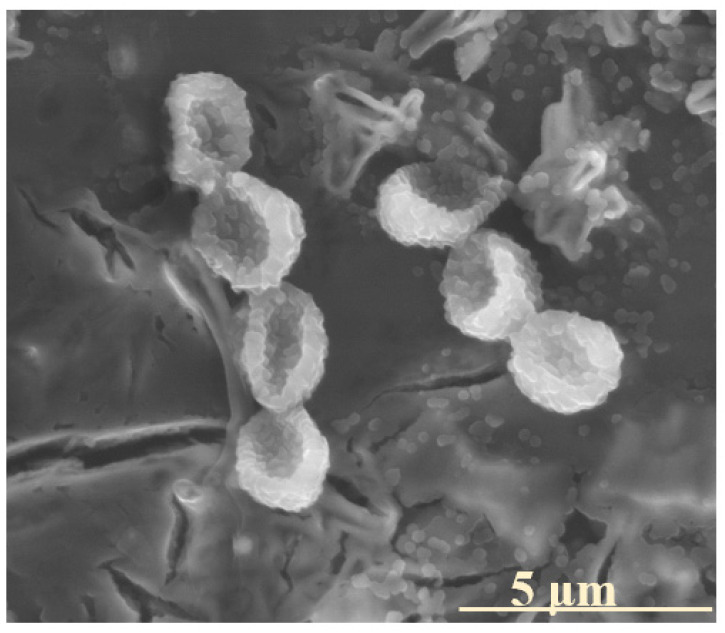
Detected biotic colonization on the surface of exemplary 0S_6PM glass after 4 months of incubation. The microphotograph was taken at 10,000× magnification.

Furthermore, the application of glasses in soil environments necessitates the consideration of biotic glass alteration, a process known to substantially contribute to the degradation of vitreous materials. Interestingly, microbial colonization was observed on the surfaces of some of the tested glasses (Figure 20). Given that microorganisms are particularly prone to adhering to mineral surfaces rich in bioavailable elements, this form of biotic weathering may serve as an additional indication of the potential utility of such materials in agricultural or environmental applications [80]. Notably, this research direction remains an active area of the authors’ ongoing scientific investigations, aiming to further explore the interplay between the glass composition–structure properties, biotic interactions, and long-term material stability.

## 3. Materials and Methods

### 3.1. Definitions

The authors would like to emphasize that, in this study, various terms describing phenomena that alter the physicochemical properties of glass (e.g., weathering, corrosion, alteration, degradation, and deterioration), despite having distinct nuances, are used interchangeably for simplicity.

Nonetheless, it is important to note that the term dissolution specifically refers to the rupture of Si-O-Si bonds and the breakdown of the silica network, whereas leaching denotes the initial degradation step involving the loss of alkali- and alkaline-earth metal ions that are ionically bonded to the silica framework—a process that precedes network dissolution [3,42]. Weathering refers to degradation resulting from prolonged exposure to water in the environment, both in the vapor and liquid states [3,42,43], while deterioration is defined as changes in the chemical composition of glass over time [44]. Moreover, the terms ‘dissolution’ and ‘corrosion’ should be used specifically when describing the transformation of glass into solely aqueous species and both solid alteration products and aqueous species, respectively.

Given its relevance to this study, the term slow-release fertilizer also requires clarification. According to the Association of American Plant Food Control Officials (AAPFCO), slow-release fertilizers are characterized by delayed nutrient availability and delivery following application, and their rate of macro-/microelement release is slower compared to that of conventional fertilizers, providing nutrients in a readily available form [45,46].

Additionally, the authors find it necessary to clarify the use of the terms ‘in vitro’ and ‘in vivo’ in the context of this study, as these are traditionally associated with medical research. Notably, in soil science, specialists consider the soil environment a ‘living entity’ due to its complex biological activity and interactions with microorganisms. Based on this perspective, we define the terms as follows:

– The term ‘in vitro’ refers here to laboratory-based experiments conducted outside the soil system, such as dissolution studies of glass fertilizers in simulated environments (e.g., solutions that mimic soil conditions);
– The term ‘in vivo’ refers here to experiments performed within a biotic soil environment, such as soil incubation studies, where glass fertilizers interact with actual soil conditions to simulate real-world scenarios.

To underscore the distinction from traditional biological applications, both terms are presented in quotation marks throughout the manuscript. This highlights their adapted use in studying glass fertilizers under controlled soil conditions.

### 3.2. Synthesis and Compositional Analysis of the Designed Glass Fertilizers

To evaluate the potential of vitreous carriers for macro- and micronutrients with prolonged-release properties, four of the most promising formulations were selected based on previous experimental findings as well as insights from related research [15,16,49,81,82,83,84].

The molar percentages of the individual oxides in each formulation are as follows (where X represents the molar percentage of SO_3_ introduced, with values of X = 0, 1, 3, or 5 mol% in this study):

– The XS_6PM system: 41SiO_2_ · 6P_2_O_5_ · 20K_2_O · 33MgO · XSO_3_;
– The XS_6PC system: 41SiO_2_ · 6P_2_O_5_ · 20K_2_O · 33CaO · XSO_3_;
– The XS_6PMC system: 41SiO_2_ · 6P_2_O_5_ · 20K_2_O · 16.5MgO · 16.5CaO · XSO_3_;
– The XS_10PM system: 41SiO_2_ · 10P_2_O_5_ · 20K_2_O · 29MgO · XSO_3_.

The above glass formulations were synthesized on a laboratory scale (~100 g) through a high-temperature melting process, utilizing raw materials of analytical-grade purity, including SiO_2_, (NH_4_)_2_HPO_4_, K_2_CO_3_, MgO, and K_2_SO_4_, all sourced from CHEMPUR (Poland). To ensure compositional accuracy, the precursor materials were meticulously weighed to a precision of 0.001 g and subsequently homogenized using a mortar and pestle. The synthesis was conducted in ceramic crucibles under ambient atmospheric conditions within an electric chamber furnace, maintaining a temperature of 1450 °C. The resulting molten glasses were subjected to rapid quenching in cold water, a process designed to achieve a fully amorphous structure while simultaneously inhibiting spontaneous crystallization [83].

The actual oxide compositions of the obtained glass samples were determined through X-ray fluorescence spectrometry (XRF) using an ARL Perform’X spectrometer (Thermo Fisher Scientific, Ecublens, Switzerland) at the accredited FerroCarbo testing laboratory. For XRF measurements, the samples—derived from homogenized glass material—were prepared in the form of fused pellets (~10 g of glass). The fusion method involved a preliminary calcination step followed by high-temperature melting in a fluxer (1100 °C) to produce analytical pearls. The XRF-determined molar contents of the individual glass components are presented in Table 3.

### 3.3. Soil Incubation Experiment (‘In Vivo’ Simulation)

To investigate the long-term nutrient release mechanisms under conditions that closely mimic real-field scenarios, yet on a laboratory scale, selected glasses representing all the synthesized compositions—incorporating 3% mol. sulfate and a base for comparison—were incubated in a soil medium. Poland, one of the largest agricultural producers in the European Union (EU), holds the largest agricultural area within the Baltic Sea drainage basin and is a key cereal producer. However, the country faces challenges related to increased soil acidity, primarily driven by inefficient and excessive nitrogen (N) fertilization, a problem also common in many European nations [84,85,86]. Therefore, as a representative medium, acidic peat was chosen, with a pH range of 3.5–4.5, and the composition (determined by X-ray fluorescence using an ARL Advant’XP spectrometer with the standard pellet method) is presented in Table 3.

Glass samples (fraction: 0.1–0.3 mm), 15 of each composition, were affixed to square pieces of carbon tape (approximately 5 × 5 mm), placed in plastic containers, and evenly mixed with the soil substrate. As-prepared specimens were then incubated in a climate chamber (Binder KMF constant climate chamber) for a 12-month period under variable climatic conditions simulating the average yearly temperature fluctuations in Poland (Table 4), with the soil watered with distilled water once a month. In the soil incubation experiments, water was not applied as a fixed volume but was instead sprayed onto the substrate until the soil surface visibly darkened—indicating the adsorption of moisture by soil particles—while avoiding the accumulation of free liquid at the bottom of the container. This corresponded to an approximate volume of a few milliliters per sample and was intended to emulate natural moisture conditions, in which water is retained in the upper soil layers before gradually percolating downwards. Such an approach ensured localized interactions between the glass particles and soil-bound water, without inducing artificial waterlogging effects.

The incubated samples were extracted from the soil substrate at various time intervals: after 2 weeks, and then monthly until the end of the experiment, to assess both the qualitative changes and alterations in the chemical compositions of the surfaces of the materials using SEM (NOVA NANO SEM 200, FEI EUROPE, Eindhoven, The Netherlands) with EDS (EDAX) in the Laboratory of Scanning Microscopy and Microanalysis at the Faculty of Materials Science and Ceramics, AGH University of Krakow. The chemical compositions of different regions on the glass surfaces were determined by EDS from at least two different spots.

To assess the applicability of the EDS data for semi-quantitative analysis, the results obtained via XRF (the pearl method) were compared with those derived from the averaged EDS analysis at three distinct points on the surface of a representative, non-altered glass sample (3S_6PM). The obtained data are presented in Table 5.

The similarity between the compared datasets suggests that the SEM-EDS analysis was highly effective for the semi-quantitative analysis, enabling the study of the surface variations on the glass throughout the ‘in vivo’ experiment. The observed deviations may have resulted from the impinging electron beam, which could have locally heated the specimens during the analysis.

## 4. Conclusions

In light of the pressing challenges facing modern agriculture—namely, the need to sustain a growing global population while the arable land availability declines and soil fertility diminishes—developing innovative strategies to replenish essential macro- and micronutrients in the soil has become imperative. Ensuring and maintaining soil health is fundamental not only for supporting plant growth and productivity but also for preserving the intricate balance of microbial communities that drive key biochemical processes within Earth’s most diverse habitats. A promising approach to addressing this challenge lies in the application of glassy fertilizers, which offer controlled and prolonged nutrient release, mitigating the risks of overfertilization and nutrient runoff—factors that can severely disrupt the biogeochemical equilibrium of soil ecosystems. Importantly, the inherent flexibility of the glassy state enables the encapsulation of a diverse array of macro- and micronutrients within an amorphous matrix, including elements that, due to their crystallo-chemical incompatibilities, cannot readily co-exist in the crystalline phase (e.g., [SiO_4_] and [SO_4_] tetrahedra [86,87,88]).

The present study centered on a soil incubation experiment of sulfate-bearing multicomponent silicate–phosphate glasses to deepen the understanding of the intricate composition–structure–property relationships within the selected composition. By focusing on the third crucial link in this chain—their real-environment behavior—this work provides insights into the mechanisms governing their interactions in soil conditions, setting the foundation for further research on their agronomic potential.

From the results obtained, the weathering behavior of the tested glasses can be effectively controlled at the compositional level. Even subtle modifications, such as altering the type of alkaline-earth network modifier or adjusting the SO_3_ and P_2_O_5_ contents, significantly influence the material’s reactivity.

MgO is known to play a dual role in glass systems, depending on its concentration, acting as either a network modifier or a former, which, in turn, increases the degree of polymerization and enhances the structural rigidity of the silicate–oxygen backbone. This reinforcement of the network architecture, evident in the XS_6PM formulation, contributed to the formation of cracks and fissures, ultimately facilitating the deeper penetration of the soil solution into the glass matrix. In contrast, the presence of CaO generally resulted in a more flexible glass network, which, in the case of the XS_6PC system, exhibited greater resistance to mechanical fracturing and a slightly reduced reactivity in the soil environment, as evidenced by the slower advancement of the reaction front into the bulk glass. The co-introduction of MgO and CaO may lead to a mixed-alkaline-earth effect, manifesting itself as an increase in the activation energy for ionic diffusion. In the formulations containing both MgO and CaO, this effect was reflected in a modest delay in the chemical reactivity compared to the other compositions.

Although indirectly, sulfate incorporation was also found to influence the material’s behavior in soil. The resulting increase in the network connectivity enhanced the structural rigidity, which, in turn, made the glasses more susceptible to mechanical damage (an effect further amplified by the positioning of the [SO_4_^2−^] species within voids of the glass matrix, inducing additional structural stresses). In the soil environment, sulfate-related effects were reflected in the deeper progression of the reaction front into the material. Notably, glasses with the highest P_2_O_5_ contents exhibited the greatest reactivity under ‘in vivo’ conditions. This not only stemmed from the significant contribution of hygroscopic [PO_4_^3−^] units but also, in all likelihood, from the high concentration of the crystallo-chemically incompatible [SiO_4_^4−^], [PO_4_^3−^], and [SO_4_^2−^] species within the framework, which destabilized the backbone structure and consequently led to the highest rates of interdiffusion reactions. Given that the propensity for glass cracking, flaking, and exfoliation directly correlates with the ion leaching intensity—by increasing the material’s contact area with the soil solution—it is evident that precise compositional modifications can be employed to regulate nutrient release rates.

Another key factor influencing the reaction kinetics at the glass–soil solution interface was the immobilization of released ions within a precipitation layer, the nature of which was strongly dependent on both the glass composition and the pH of the surrounding medium. The registered effect is of utter importance given the intended materials’ application: as the fraction of nutrients not directly utilized by plants accumulates in the soil solution, excess ions are expected to precipitate as secondary phases on the glass surface. Importantly, the composition-dependent nature of these secondary phases dictates their subsequent dissolution rates, thereby influencing the long-term availability of both micro- and macronutrients in acidic soil environments.

A particularly crucial aspect of this study, from the perspective of utilizing these materials as glassy carriers of essential nutrients, was determining the sequential release of the matrix constituents into the soil medium. The findings revealed that K^+^ ions, possessing low cationic field strength and resulting in a strong tendency to diffuse toward the glass surface, were leached preferentially, followed by the release of alkaline-earth cations and [PO_4_^3−^] and [SO_4_^2−^] species, whereas the silicon–oxygen backbone remained largely intact over the duration of the experiment. Notably, in all the tested cases, the [SO_4_^2−^] species were released in the early stages of the incubation, likely due to their high solubility in acidic conditions. Furthermore, the dissolution was found to be selective, with the formation of a silica-rich crust that retained residual, unleached ions, confirming an incongruent dissolution mechanism.

Despite the controlled nature of this closed-system study, the findings successfully captured the complex and interdependent processes occurring at the soil–glass interface, emphasizing the critical roles of the soil pH, the glass composition, and environmental variability in governing glass–soil interactions. The acidic nature of the peat dictated the primary reaction mechanisms, favoring ion exchange, while limited water availability led to rapid solution saturation with leached elements, promoting the precipitation of secondary phases. Notably, the identity of these crystallized phases was strongly influenced by both the solution pH and the interplay between the glass and soil compositions, whereas surface characteristics—such as roughness, microdefects, and compositional heterogeneities—played a decisive role in governing the timing and localization of the glass–solution interactions.

These findings highlight the multifactorial nature of glass degradation, where the sequence of events and the predominance of specific processes remain difficult to predict with certainty, underscoring the necessity of considering unforeseen phenomena when assessing glass–soil interactions, as well as the importance of further investigations under more dynamic and complex environmental conditions.

### Final Remarks

The findings from this study provide valuable insights into the long-term alteration mechanisms of glass, which can aid in the development of more accurate predictive models for understanding the complex behavior of glass materials under various environmental conditions. Furthermore, these insights may prove crucial in the design of advanced vitreous fertilizers, optimizing their nutrient release profiles while minimizing their environmental impact.

## Figures and Tables

**Table 1:** Summary of surface characteristics of glass samples after soil incubation, detailing the types of crystalline phases identified and the extent of the surface fragmentation.

Month	0.5	1	2	3	4	5	6	7	8	9	10	11	12
0S_ 6PM	M	I	I	N	M	H	E	H	E	H	E	H	H
	**MP**	**KMP**	**MP**	**MP**	**MP**	**MP**	**MP**	**MP**	**MP**	**MP**	**MP**	**MP**	**MP**
	**Localized leaching**					**Intense leaching**							
3S_ 6PM	I	N	I	M	M	M	M	E	E	E	H	H	E
	-	**KMP**	**MP**	**MP**	**MP**	**MP**	**MP**	**MP**	**MP**	**MP**	**MP**	**MP**	**MP**
	**Localized leaching**			**Intense leaching**									
0S_ 6PC	N	I	I	I	M	N	M	M	E	M	H	H	H
	**CP small precipitate deposit**										**Thinning of the CP deposit**		
	**Localized leaching**						**Intense leaching**						
3S_ 6PC	M	I	M	E	H	E	E	E	H	H	E	E	E
	**CaP small precipitate deposit**										**Thinning of the CaP deposit**		
	**Localized leaching**				**Intense leaching**								
0S_ 6PMC	N	M	N	M	M	M	H	H	E	E	H	H	E
	-	**CP**	MP	MP **CC**	**CP** **CC**	**CC**	**CP** **CC**	**CP**	**CP**	**CP**	**CP**	**CC**	**CP**
	**Localized leaching**						**Intense leaching**						
3S_6PMC	I	I	I	*	*	E	E	E	E	E	E	E	E
	-	**CC**	MP **CC**	**KMP** **CC**	**KMP** **CC**	**KMP** **CC**	**CP**	**CP**	**CP**	**CP**	-	-	-
	**Localized leaching**					**Intense leaching**							
0S_10PM	E	E	E	E	E	E	C	C	M	H	H	C	M
	**KMP**	**KMP**	**KMP**	**KMP**	**KMP**	**KMP**	-	-	-	-	-	-	-
	**Localized leaching**					**Intense leaching**							
3S_10PM	E	E	E	E	E	C	C	C	C	C	C	C	C
	**KMP**	**KMP** **MP**	**KMP**	**KMP**	**KMP**	**KMP** **MP**	**MP**	**MP**	**MP**	**MP**	**MP**	**MP**	**MP**
	**Localized leaching**					**Intense leaching**							

Legend: Types of crystals suggested by the semi-quantitative SEM-EDS analysis: MP—magnesium phosphate, KMP—potassium magnesium phosphate, CP—calcium phosphate, CC—calcium carbonate. Surface condition (fragmentation/cracking): N: no visible cracks—no shading, I: isolated cracks—light pink, M: moderate concentration of cracks—light red, H: high concentration of cracks—red, E: cracks covering the entire surface of all fragments—dark red, C: complete fragmentation of glass pieces—gray. Shaded fields indicate areas where dense layers of crystallites uniformly covered the glass surface. * Note that the evaluation of the surface condition at 3 and 4 months of incubation was not possible due to the dense layer of precipitation products, which obscured detailed assessment.

**Table 2:** Chemical composition of the soil substrate used (mol. %).

Soil Composition, mol.%			
SiO_2_	38.64	P_2_O_5_	0.88
CaO	27.72	Na_2_O	0.62
SO_3_	8.94	MnO	0.21
Fe_2_O_3_	6.28	Br	0.20
Al_2_O_3_	5.02	ZnO	0.20
Cl	3.80	PbO	0.19
MgO	3.09	SrO	0.13
K_2_O	2.68	ZrO_2_	0.10
		TiO_2_	1.30

**Table 3:** Measured compositions of the synthesized specimens. Registered minor discrepancies between the expected and actual amounts of glass components are attributed to inherent measurement uncertainties and potential volatilization during the heating process.

No.	SiO_2_	P_2_O_5_	K_2_O	MgO	CaO	SO_3_
0S_6PM	41.42 (±0.40)	6.75 (±0.09)	20.42 (±0.00)	30.17 (±0.45)	-	-
1S_6PM	37.39 (±0.41)	5.96 (±0.09)	19.53 (±0.00)	31.72 (±46)	-	0.84 (±0.12)
3S_6PM	40.00 (±0.41)	6.92 (±0.09)	20.63 (±0.00)	32.67 (±0.46)	-	1.84 (±0.14)
5S_6PM	41.60 (±0.40)	6.49 (±0.40)	17.69 (±0.00)	33.44 (±0.40)	-	1.59 (±0.40)
0S_6PC	39.11 (±0.44)	6.29 (±0.09)	18.88 (±0.00)	-	33.63 (±0.71)	-
1S_6PC	38.28 (±0.45)	6.14 (±0.10)	17.21 (±0.00)	-	35.79 (±0.73)	1.00 (±0.16)
3S_6PC	37.90 (±0.45)	6.46 (±0.09)	18.40 (±0.00)	-	35.12 (±0.72)	2.05 (±0.13)
5S_6PC	43.25 (±0.45)	6.10 (±0.01)	15.03 (±0.00)	-	37.42 (±0.72)	1.39 (±0.15)
0S_6PMC	39.91 (±0.41)	6.33 (±0.09)	18.40 (±0.00)	15.46 (±0.34)	18.16 (±0.44)	-
1S_6PMC	38.20 (±0.42)	6.33 (±0.09)	19.59 (±0.00)	15.53 (±0.33)	18.09 (±0.56)	0.95 (±0.12)
3S_6PMC	38.08 (±0.09)	6.64 (±0.09)	18.92 (±0.00)	16.57 (±0.34)	20.15 (±0.45)	1.76 (±0.15)
5S_6PMC	41.70 (±0.42)	6.46 (±0.09)	16.41 (±0.00)	16.32 (±0.35)	20.89 (±0.56)	1.40 (±0.14)
0S_10PM	38.11 (±0.40)	11.01 (±0.08)	21.56 (±0.00)	29.12 (±0.44)	-	-
1S_10PM	39.00 (±0.42)	10.89 (±0.09)	19.95 (±0.00)	28.98 (±0.50)	-	0.25 (±0.05)
3S_10PM	39.37 (±0.42)	11.02 (±0.09)	21.14 (±0.00)	29.35 (±0.47)	-	0.83 (±0.10)
5S_10PM	42.29 (±0.41)	11.48 (±0.08)	22.29 (±0.00)	24.71 (±0.46)	-	0.48 (±0.07)

**Table 4:** Characterization of simulated seasonal climatic conditions.

Simulated Seasons	Spring (Mar–May)	Summer (Jun–Aug)	Autumn (Sep–Nov)	Winter (Dec–Feb)
Day/night average temperature (°C)	13.5/3.8	23.1/13.8	13.2/5.5	1.8/−4.2
Average humidity (%)	71	71	79	- *

* The average humidity data for the winter period could not be simulated in the experiment due to chamber limitations, as the chamber was unable to control air humidity at temperatures below 0 °C.

**Table 5:** Comparison between the chemical composition of the selected sample obtained via XRF and SEM-EDS method.

Data Origin	SiO_2_	P_2_O_5_	K_2_O	MgO	SO_3_
XRF	40.00 (±0.40)	6.92 (±0.08)	20.63 (±0.00)	32.67 (±0.59)	1.84 (±0.14)
SEM-EDS	44.64 (±0.34)	7.70 (±0.23)	19.15 (±1.96)	27.02 (±2.17)	1.49 (±0.45)

## Data Availability

The data presented in this study are available upon request from the corresponding author.
